# Supplementary material for: Predictors of Early Response, Flares, and Long-Term Adverse Renal Outcomes in Proliferative Lupus Nephritis: A 100-Month Median Follow-Up of an Inception Cohort
Source: J Clin Med. 2022 Aug 26;11(17):5017. doi: 10.3390/jcm11175017 (PMC9457419; doi:10.3390/jcm11175017)

Supplemental Table S1. Differences in baseline characteristics between the two major induction treatment groups (mycophenolic acid versus cyclophosphamide).

**Baseline characteristics**	**Mycophenolic acid group (N=27)**	**Cyclophosphamide group (N=69)**	**p-value**
Age (yr) median(IQR)	31(22)	26(17)	0.25
Sex (M-F)N/%	5/18-22/82	14/20-55/80	1
Time from SLE diagnosis to LN (years)median(IQR)	7.5(8)	3(4.5)	**0.02**
SLEDAI score median(IQR)	10(5)	12(2)	**0.01**
Low C3 N/%	14/56	51/91	**0.001**
Low C4 N/%	11/44	44/78	**0.002**
Positive anti-dsDNA N/%	18/78	43/79	0.9
Proteinuria (g/24h) mean ± SD	1.3 ±2.7	3 ±3.6	**0.009**
• Proteinuria >3g/d N/%	9 / 34	37 / 53	**0.004**
• Proteinuria 1-3g/d N/%	6 / 22	23 / 33	
• Proteinuria <1g/d N/%	12 / 44	9 / 14	
Active urine sediment N/%	22/81	67/97	**0.01**
Hypertension N/%	6/22	21/30	0.4
Serum albumin (g/dl) median(IQR)	3.7(1)	2.8(0.7)	**0.001**
Serum Cr(mg/dl) median(IQR)	0.8(0.4)	0.8(0.8)	0.56
eGFR (ml/min/1.73m^2) mean ± SD	100 ± 39	95 ± 66	0.67
• eGFR >60 N/%	23/85	49/70	0.09
• eGFR 30-60 N/%	4/15	10/15	
• eGFR <30 N/%	-	10/15	
LN class			
• III N/%	13/48	13/19	**<0.01**
• IV N/%	6/22	40/58	
• III + V N/%	6/22	2/3	
• IV + V N/%	2/7	14/20	
Number of crescents median(IQR)	1(2)	3(5)	**0.007**
Activity indexmedian(IQR)	7(5.5)	11(6)	**<0.001**
Chronicity index median(IQR)	2(3)	2(2)	0.67
Interstitial fibrosis/tubular atrophy*			0.73
• <25% N/%	23 / 85	59 / 88	
• >25% N/%	4 / 15	8 / 12	
Duration of treatment (months) median(IQR)	38.5(38)	43(45)	0.3

eGFR: estimated glomerular filtration rate using the CKD-EPI formula, SLEDAI: systemic lupus erythematosus disease activity index, anti-ds DNA: antibodies against double stranded DNA
*****refers to percentage of renal cortex involved by interstitial fibrosis and tubular atrophy

Supplemental Table S2. Predictors of complete response at 3-6-9-12-18-24 months.

**Variables**	**Complete Remission**		
	**OR**	**95%Cis**	**p-value**
**3 months**<br>Univariate models			
**Proteinuria at diagnosis (g/day) <1.5**	**9.4**	**3.16, 27**	**<0.001**
**6 months**<br>Multivariate model			
**eGFR at diagnosis(ml/min/1.73m^2)**<br>**>60**	2.85	0.85, 9.5	0.08
**Proteinuria at diagnosis (g/day) <1.5**	**5.3**	**1.7, 16.5**	**0.004**
**9 months**<br>Multivariate model			
**eGFR at diagnosis(ml/min/1.73m^2)**<br>**>60**	**4.04**	**1.22, 13**	**0.02**
**Proteinuria at diagnosis (g/day) <1.5**	**3.7**	**1.18, 1.17**	**0.02**
**12 months**<br>Multivariate model			
**eGFR at diagnosis (ml/min/1.73m^2)**	1.68	0.53, 5.27	0.37

**>60**			
**Proteinuria at diagnosis (g/day) <1.5**	**16.9**	**2.12, 134**	**0.008**
**Number of Crescents**	0.92	0.81, 1.03	0.18
**18 months** Univariate models			
**Proteinuria at diagnosis (g/day) <1.5**	**5.24**	**1.42, 19**	**0.01**
**24 months** Univariate models			
**Proteinuria at diagnosis (g/day) <1.5**	**4**	**1.08, 15**	**0.03**

Supplemental Table S3. Response (CR or PR) at different time points as predictors of flare.

Categories	Univariate Models	
	**OR**	**95 CIs (p-value)**
**Response at 3months**		
PR or CR	Reference group	
None	1.05	0.41, 2.7 (0.9)
**Response at 6months**		
PR or CR	Reference group	
None	1.25	0.45, 3.4 (0.66)
**Response at 9months**		
PR or CR	Reference group	
None	2.2	0.65, 7.8 (0.19)
**Response at 12months**		
PR or CR	Reference group	
None	**3.8**	**1.03, 14 (0.04)**
**Response at 18months**		
PR or CR	Reference group	
None	**4.9**	**1.4, 17 (0.01)**
**Response at 24months**		
PR or CR	Reference group	
None	**6.6**	**1.2, 34 (0.02)**

CR:complete response, PR: partial response

Supplemental Table S4. Statistical tests examining correlation between independent predictors of risk of flare.

	**LN class (III, IV, III/IV+V)**	**Induction Treatment (CYC, MPA)**	**Age (years)**	**Proteinuria at 12m (>0.8 vs<0.8 g/day)**	**Time to Either Remission (months)**
**Proteinuria at diagnosis (>2 vs <2 g/day)**	Chi^2 (p<0.001) Cramer's V=0.44	Chi^2 (p=0.006) Cramer's V=- 0.28	Mann−Whitney *U* (p=0.12)	Chi^2 (p=0.01) Cramer's V=0.28	Mann−Whitney *U* (p=0.01)
**LN class (III, IV, III/IV+V)**	-	Chi^2 (p=0.003) Cramer's	Anova (p=0.42)	Chi^2 (p=0.35) Cramer's	Anova (p=0.65)

		V=0.34		V=0.16	
**Induction Treatment (CYC, MPA)**	-	-	Mann−Whitney *U* (p=0.5)	Chi^2 (p=0.37) Cramer's V= - 0.1	Mann−Whitney *U* (p=0.19)
**Age (years)**	-	-	-	Mann−Whitney *U* (p=0.1)	Spearman Correlation Coefficient r_s= 0.14
**Proteinuria at 12m (>0.8, <0.8 g/day)**	-	-	-	-	Mann−Whitney *U* ($p<0.001$)

Supplemental Table S5. Renal function at presentation, response to treatment and time to renal failure in patients with ESRD.

Patients with ESRD	GFR at LN diagnosis (ml/min/1.73m^2)	Renal Response	Time to ESRD (months)
1	11	NR	0
2	110	PR	70
3	49	CR	147
4	34	CR	180
5	53	NR	55
6	22	NR	9
7	91	NR	4
8	19	PR	76
9	51	CR	159
10	12	CR	183
11	19	NR	4
12	15	NR	5

eGFR: estimated glomerular filtration rate using the CKD-EPI formula, CR:complete response, PR: partial response, NR: no response, ESRD: end stage renal

Supplemental Table S6. Responses at different time points as predictors of adverse renal outcome.

**Categories**	**Univariate Models**	
	**OR**	**95 CIs (p-value)**
**Response at 3months**		
PR or CR	Reference group	
None	2.2	0.82, 6.3 (0.11)
**Response at 6months**		
PR or CR	Reference group	
None	**6.4**	**2.16, 19 (0.001)**
**Response at 9months**		
PR or CR	Reference group	
None	**5.5**	**1.5, 20 (0.01)**
**Response at 12months**		
PR or CR	Reference group	
None	**17.4**	**4, 75 (<0.001)**
**Response at 18months**		
PR or CR	Reference group	
None	**10.7**	**2.9, 39 (<0.001)**
**Response at 24months**		
PR or CR	Reference group	
None	**10.5**	**2.3, 47(0.002)**

Supplemental Table S7. Statistical tests examining correlation between independent predictors of risk of adverse renal outcome.

	**eGFR at diagnosis (>60, <60 ml/min/1.73m^2)**	**Interstitial fibrosis/Tubular atrophy (<25%, >25%)**	**Proteinuria at 12m (>0.8, <0.8 g/day)**	**Time to Either Remission (months)**
**Proteinuria at diagnosis (>1.5<1.5g/day)**	Chi^2 (p=0.01) Cramer's V=0.23	Chi^2 (p=0.51) Cramer's V=-0.06	Chi^2 (p=0.008) Cramer's V=0.29	Mann−Whitney *U* (p=0.02)
**eGFR at diagnosis (>60, <60 ml/min/1.73m^2)**	-	Chi^2 (p<0.001) Cramer's V=0.39	Chi^2 (p=0.08) Cramer's V=0.19	Mann−Whitney *U* (p=0.03)
**Interstitial fibrosis/Tubular atrophy (<25%, >25%)**	-	-	Chi^2 (p=0.54) Cramer's V=0.06	Mann−Whitney *U* (p=0.24)
**Proteinuria at 12m (>0.8, <0.8 g/day)**	-	-	-	Mann−Whitney *U* (p<0.001)

Supplementary Figure S1: Kaplan-Meier survival estimates of probability for response according to [a] proliferative lupus nephritis class [b] treatment with cyclophosphamide versus MPA/MMF

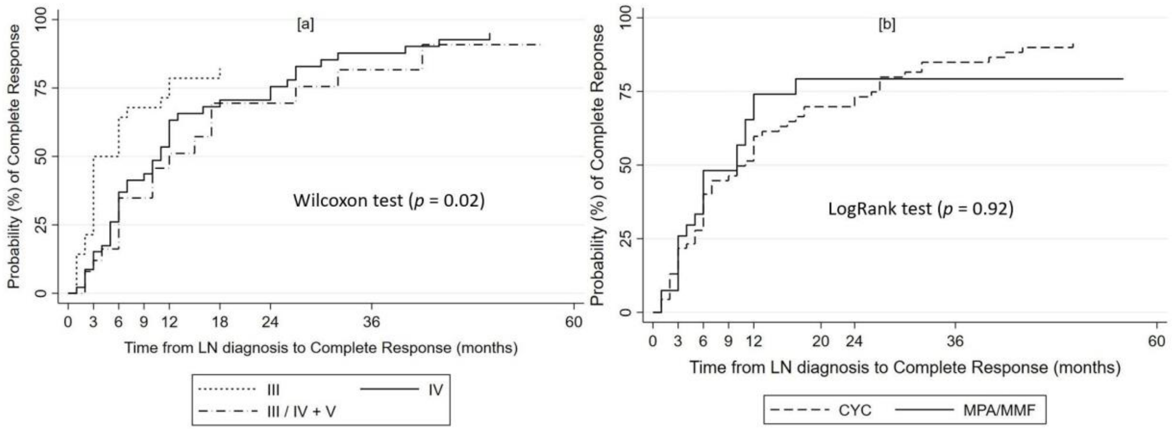

Supplement: Supplementary file 1 [file jcm-11-05017-s001.zip › jcm-1862074-supplementary.pdf]
